# Site‐specific recombinase genome engineering toolkit in maize

**DOI:** 10.1002/pld3.209

**Published:** 2020-03-09

**Authors:** Jon P. Cody, Nathaniel D. Graham, Changzeng Zhao, Nathan C. Swyers, James A. Birchler

**Affiliations:** ^1^ Division of Biological Sciences University of Missouri Columbia MO USA

**Keywords:** *Agrobacterium*, bombardment, genetic engineering, maize, recombinases

## Abstract

Site‐specific recombinase enzymes function in heterologous cellular environments to initiate strand‐switching reactions between unique DNA sequences termed recombinase binding sites. Depending on binding site position and orientation, reactions result in integrations, excisions, or inversions of targeted DNA sequences in a precise and predictable manner. Here, we established five different stable recombinase expression lines in maize through *Agrobacterium*‐mediated transformation of T‐DNA molecules that contain coding sequences for Cre, R, FLPe, phiC31 Integrase, and phiC31 excisionase. Through the bombardment of recombinase activated DsRed transient expression constructs, we have determined that all five recombinases are functional in maize plants. These recombinase expression lines could be utilized for a variety of genetic engineering applications, including selectable marker removal, targeted transgene integration into predetermined locations, and gene stacking.

## 1 INTRODUCTION

Successful transfer and expression of foreign DNA in plant cells through the process of transformation was achieved over 30 years ago (Bevan, Flavell, & Chilton, 1983; Fraley et al., 1983; Herrera‐Estrella et al., 1983; Murai et al., 1983). Since then, genetic engineering has been used as a major tool in a worldwide effort to increase agricultural productivity and performance in staple crops, such as rice, soybean, and maize. Future challenges of sustaining a growing population and adapting to shifting environmental conditions create a need for advancements in crops beyond what is possible using conventional genome engineering methods, which randomly integrate DNA into host genomes using *Agrobacterium tumefaciens* or biolistic bombardment. The absence of targeting machinery, together with low DNA carrying capacity on commonly used plasmid vectors, limits researchers to a few genes in a single transformation experiment, a process that takes 6 months—1 year in most plant species. This creates a bottleneck when applying known biological systems, such as biosynthetic pathways or disease resistance clusters, to create healthy and affordable food supplies (Altpeter et al., 2016). Multiple transgenes in different loci become increasingly difficult to maintain in subsequent generations due to independent segregation in meiosis, requiring a labor‐intensive and time‐consuming process of introgression into agronomically significant cultivars. Additionally, recovered events from plant transformations exhibit differences in expression level, copy number, and can possibly interfere with endogenous gene function; therefore, it is necessary to screen each event for optimal insertion locations, eliminating upwards of 90% of T_0_ plants under commercial parameters (Anand & Jones, 2018). Recent developments of precise genome editing techniques, such as zinc‐finger nucleases (ZFN) (Bibikova, Beumer, Trautman, & Carroll, 2003; Carroll, 2011), transcription activator‐like effector nucleases (TALENs) (Christian et al., 2010; Bogdanove and Voytas 2011), and CRISPR‐Cas9 (Jinek et al., 2012), create promising avenues for targeted modifications; however, they have a low frequency of transgene targeting. Alternatively, the use of site‐specific recombinases could enable efficient strategies for targeted modifications and possibly give researchers more control over established transgenic lines.

**Figure 1:**
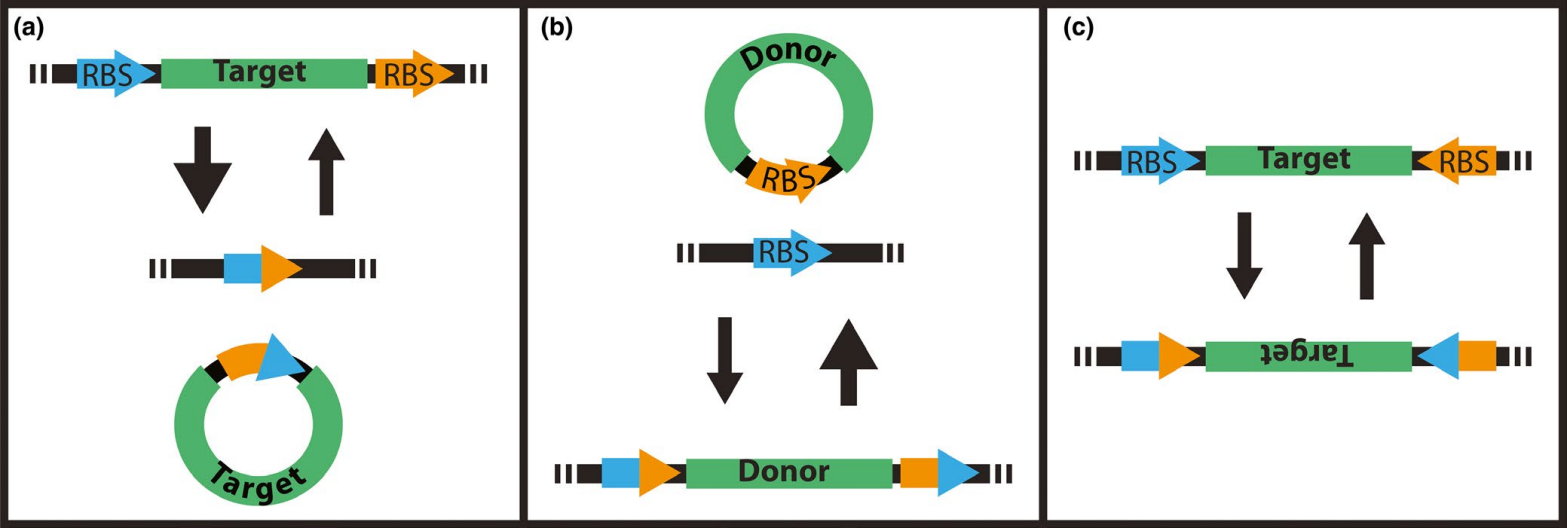
Site‐specific recombinase modifications: Depending on recombinase binding site (RBS) position and orientation, recombinase reactions will result in (a) excisions, (b) integrations, or (c) inversions of specific sequences, such as selectable markers and genes of interest. DNA used for targeted integrations must be in a circular conformation to avoid fragmentation of engineered sequences. Arrows indicate directionality of the recombinase reaction. Large arrows indicate favorability of intramolecular reactions. RBS sites are shown in blue and orange arrowheads. Recombination events exchange portions of the RBS reciprocally. (a) RBSs flanking a target are recombined to remove the sequences between the RBSs. (b) A circular DNA molecular carrying a RBS is introduced to cells expressing the respective recombinase and with a target RBS present. Recombination between the RBSs will place the introduced DNA into the pre‐existing RBS. (c) When RBSs flank a sequence in inverted orientation, introduction of the appropriate recombinase will invert the sequences between the sites, which can be used for gene activation or inactivation

Site‐specific recombinases are a class of specialized enzymes that catalyze strand‐switching reactions between two specific DNA sequences termed recombinase binding sites (RBS). While they are most prevalent in phage, bacterial, and lower eukaryotic organisms, serving specific biological functions, recombinases are active in a variety of heterologous environments, including in vitro conditions. The reactions involve the following: (a) recognition and binding of recombinase dimers to binding sites, (b) formation of a synaptic complex between two bound sites, (c) recombinase‐mediated strand exchange and fusion events, (d) disassembly of the synaptic complex (Grindley, Whiteson, & Rice, 2006). Depending on position and orientation of the sites, reactions will result in integrations, excisions, or inversions of intervening DNA sequences in precise and predictable manners (Figure 1). While the most widely used and described recombinases are Cre (causes recombination) and FLP (flippase), there exists a collection of enzymes that are all unique in terms of origin and sequence recognition; however, all fall into one of the two specific families, reflecting the recombinase amino acid group that catalyzes strand‐switching reactions.

The Tyrosine family of recombinases includes Cre (Sauer & Henderson, 1989), FLP (Golic & Lindquist, 1989), and R (Onouchi et al., 1991), which bind to identical recombinase binding sites *lox, FRT,* and *RS*, respectively. Since Tyrosine recombinases catalyze strand‐switching reactions between identical sites (Table S1), the reaction is bidirectional, favoring intramolecular over intermolecular recombination. The Serine family of recombinases includes phiC31 Integrase and phiC31 excisionase (Thorpe & Smith, 1998), which binds to non‐identical sites *attB/attP* and *attL/attR*, respectively. Recombination reactions between non‐identical sequences create hybrid binding sites that are unrecognized by recombinases mediating the strand‐switching reaction. PhiC31 Integrase forms a synaptic complex between *attB* and *attP* binding sites and induces a strand‐switching reaction to form *attL* and *attR*, which are unrecognizable in the absence of the helper protein phiC31 excisionase. If excisionase helper proteins are expressed in the presence of phiC31, recombination reactions between attL and attR favor the reverse direction.

The first application using Cre to excise a stably integrated selectable marker gene from tobacco occurred in the early 90s (Dale & Ow, 1990). Since then, recombinases have been used in a number of plant systems including rice (Hoa, Bong, Huq, & Hodges, 2002; Hu et al., 2007; Radhakrishnan and Srivastava, 2005), tobacco (Albert, Dale, Lee, & Ow, 1995; Dale & Ow, 1991), wheat (Srivastava, Anderson, & Ow, 1999), tomato (Stuurman, Vroomen, Nijkamp, & Haaren, 1996), barley (Kapusi, Kempe, Rubtsova, Kumlehn, & Gils, 2012), soybean (Li et al., 2009), *Arabidopsis* (Hong, Lyznik, Gidoni, & Hodges, 2000; Thomson, Chan, Thilmony, Yau, & Ow, 2010; Vergunst & Hooykaas, 1998), and maize (Anand et al., 2019; Kerbach, Lörz, & Becker, 2005; Lyznik, Rao, & Hodges, 1996; Srivastava & Ow, 2001; Zhang et al., 2003). In these studies, recombinase enzymes have been used for the purpose of selectable marker removal, transgene targeting to predetermined locations, or resolution of multiple transgene concatemers. While most studies use bidirectional recombinases, the use of mutant binding sites or transient expression forces unidirectional activity and fixes desired modifications, such as targeted integrations (Albert et al., 1995); however, integration stability using double‐mutant sites with FLP/*FRT* has not been successful, and R/*RS* has no known mutant sites (Wang, Yau, Perkins‐Balding, & Thomson, 2011). Alternatively, a strategy referred to as recombinase‐mediated cassette exchange (RMCE) has been used successfully to direct transgenes to predetermined locations using FLP/*FRT* and R/*RS* systems (Anand et al., 2019; Ebinuma, Nakahama, & Nanto, 2015).

While much work has been performed using recombinases in plant systems, researchers using these enzymes are required to create stable expression lines to carry out modifications; otherwise, they must rely on strategies of transient expression, a process that uses the conventional transformation procedure to deliver recombinase expression cassettes via *Agrobacterium* or biolistic bombardment. In this work, we have established and demonstrated the functionality of five different recombinase systems in maize: Cre, R, FLPe, phiC31 Integrase, and phiC31 excisionase, which can be used as tools to carry out genome modifications.

## 2 MATERIALS AND METHODS

### 2.1 Plant transformation constructs

Cre recombinase expression vector, pTFUbiCre, was previously described by Gaeta et al. (2013). pTFUbiCre contains a maize ubiquitin I promoter (Ubi) driving the expression of Cre, followed by a nopaline synthase termination sequence (NOSt). Tissue culture selection is provided by a bialaphos resistance marker, expressed by the CaMV 35S promoter from the Cauliflower mosaic virus. A *SmR* gene positioned on the vector backbone confers resistance to spectinomycin and streptomycin in *E. coli* or *Agrobacterium* cell cultures (Figure S1a).

Coding sequences for R recombinase were provided by Jan Schaart of Wageningen University & Research in Wageningen, Netherlands (Schaart, Krens, Pelgrom, Mendes, & Rouwendal, 2004). The R sequence was synthesized by GenScript and modified to contain a SV40 nuclear localization signal (NLS) on the 3′ end. The R gene was cloned into pTFUbiCre using restriction endonucleases *Xma*I and *Sac*I, which replaced Cre with R to create pTF‐R (Figure S1b).

Enhanced version of FLP, FLPe, was provided by Vibha Srivastava of the University of Arkansas (Akbudak & Srivastava, 2011). Ubi‐FLPe‐NOSt‐NOSt coding sequence from pAA8 was PCR amplified using forward and reverse primers 5′–TGTGGAATTGTGAGCGGATA–3′ and 5′–TGCCACCTGACGTCTAAGAA–3′, respectively. PCR products were digested and cloned into transformation vector pZY101 using *Hin*dIII and *Eco*RI restriction endonucleases to produce pTF‐FLPe (Figure S1c).

The phiC31 Integrase construct, pICH13130, was provided by Mario Gils of Leibniz‐Institut fur Pflanzengenetik und Kulturpflanzenforschung (IPK), Gatersleben, Germany (Rubtsova et al., 2008). pICH13130 contains a Ubi promoter driving expression of a modified phiC31 Integrase gene that includes a 300 bp intron insert from *Petunia hybrida* and a SV40 NLS on the 3′ end. Ubi‐phiC31 Integrase‐NOSt was cloned into the binary transformation vector pTF101.1 to produce pTFPhiC31 (Figure S1d).

The phiC31 excisionase construct, pCS‐kRI, was provided by Michele Calos of Stanford University School of Medicine (Farruggio, Chavez, Mikell, & Calos, 2012). Since phiC31 excisionase is a modifying protein that binds to phiC31 Integrase, enabling the recombination of attL and attR binding sites, both Integrase and excisionase must be present on the same vector. pCS‐kRI contains phiC31 excisionase fused to phiC31 Integrase via a P2A peptide. The phiC31 excisionase/Integrase coding sequence was cloned into plasmid pWY56c, which contains nested multiple cloning site (MCS) between a Ubi promoter and a NOSt sequences. The Ubi‐phiC31 excisionase/Integrase‐NOSt fragment was subsequently cloned into the transformation vector pZY101 to form pZY56IntExc (Figure S1e). All constructs listed above were verified using Sanger sequencing.

### 2.2 DsRed test constructs

**Figure 2:**
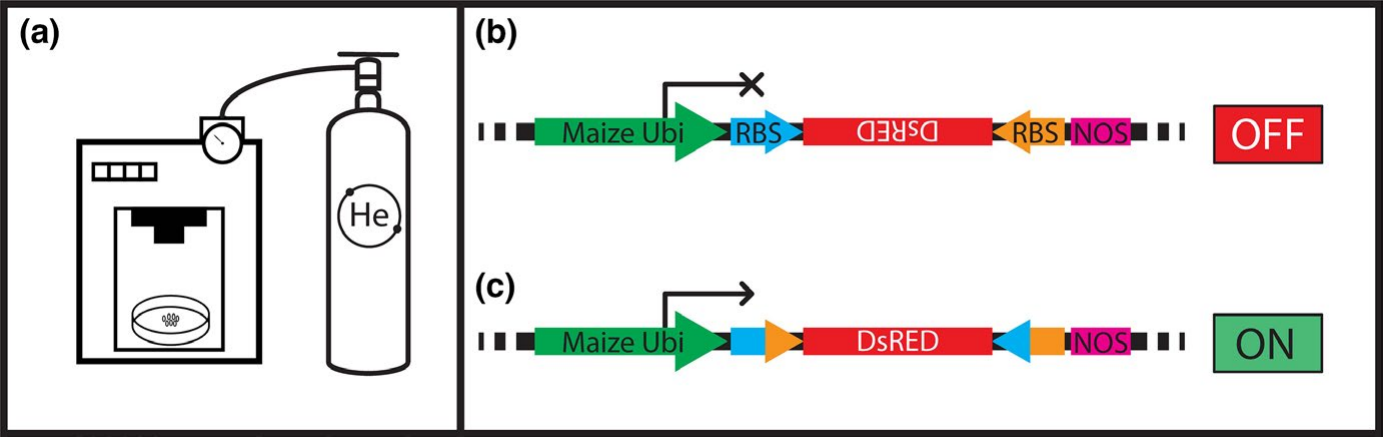
Testing recombinase activity in Maize: (a) 1000/He Particle Delivery System (Bio‐Rad) was used to deliver a DsRed expression construct into immature embryos (9–12 days after pollination) of different recombinase expression lines and Hi‐II controls. (b) Anti‐DsRed expression construct was used to test recombinase activity. Constructs contain a maize ubiquitin‐1 promoter (Ubi) behind an inverted DsRED gene, which is flanked by recombinase binding sequences (RBS) unique to each recombinase tested. (c) After bombardment of an inverted DsRed construct, recombinase enzymes bind to respective sites and catalyze an inversion reaction, activating DsRed expression

The plasmid pWY56c, which contains a nested MCS between Ubi and NOSt sequences, was used in the production of all *DsRED* test constructs. As shown in Figure 2b, each test construct contains a *DsRED* gene, flanked by oppositely oriented recombinase binding sites, and inverted with respect to a maize Ubi promoter. These sequences (Table S1), RBS‐*DsRed*‐RBS, were each synthesized and cloned into pWY56c by GeneWiz to produce pCre‐AntiDsReD, pR‐AntiDsRed, pFLPe‐AntiDsRed, pIntegrase‐AntiDsRed, and pexcisionase‐AntiDsRed. It is important to note that pCre‐AntiDsReD contains mutant RBS sites of *lox66* and *lox*71 (Albert et al., 1995).

### 2.3 Plant transformation

Each recombinase expression vector was transformed into EHA105 *Agrobacterium* cells (provided by Stanton B. Gelvin, Purdue University) by electroporation and selected LB agar plates using rifampicin (30 μg/ml), spectinomycin (100 μg/ml), and streptinomycin (100 μg/ml) antibiotics. Single colonies were suspended in 5 ml of LB medium, containing the appropriate antibiotics, and grown for 24–48 hr at 28°C, shaking at 250 rpm. Glycerol stocks of each culture were stored at −80°C and used to create *Agrobacterium* growth plates used in the transformation procedure.

Immature embryo explants were obtained from crosses of Hi‐II A × Hi‐II B lines (Armstrong & Green, 1985). Embryos were collected 9–12 days after pollination at a length of ~1.2–1.5 mm. Immature embryos were sterilized and inoculated with *Agrobacterium* EHA105 infection media (OD_600_ = 0.3–0.5) for 3–4 hr. Embryos were transferred to cocultivation plates and placed at 25°C for 3–4 days. Infected embryos were transferred to callus induction plates and placed at 28°C for 11–12 days. Type II callus from callus induction plates were transferred to selection plates containing 3 μg/ml bialaphos and switched to new nutritional media every 2 weeks until resistant cell masses were formed, which can take up to 12 weeks. Root regeneration and shoot regeneration were carried out sequentially, taking ~3 weeks each. For a full description of the plant transformation process, including a complete list of media reagents see Lee and Zhang (2013). For each transformation experiment, a single event was chosen to regenerate, propagate, and test for recombinase functionality, which for each example was successful.

### 2.4 PCR analysis of T_0_ transgenics

Genomic DNA from leaf tissue was extracted from T_0_ transgenic plants using the method described (Leach, McSteen, & Braun, 2016). PCR on extracted genomic DNA was performed using standard conditions of 35 cycles: 30 s denature at 95°C, 30 s annealing at 60°C, and Xs extension at 72°C. Primer sets used for the screening process are listed in Table S2 and are all designed to amplify a fragment of approximately 500 bp.

### 2.5 Embryo bombardment

Particle bombardment was performed on immature embryo explants of Hi‐II × homozygous recombinase expression and Hi‐II control lines, harvested 10–12 days after pollination. Embryos' length was approximately 1.5–2 mm in length. After extraction from ears, embryo explants were placed on N6 media for 3–5 days prior to bombardment (Songstad, Armstrong, Petersen, Hairston, & Hinchee, 1996). Four hours prior to bombardment, embryos were transferred to osmotic media. Then, 0.6‐μm gold particles (Bio‐Rad) were prepared following the steps outlined by Iowa State Plant Transformation Facility (Frame et al., 2000; Sanford, Smith, & Russell, 1993). Approximately 1 μg of each recombinase test construct was used in the 120‐μl gold mixture. 10 μl of the gold particle suspension was pipetted onto macrocarriers (Bio‐Rad) and allowed to dry in a hood. After drying, another 10 μl of the gold particle suspension was added again, equating to ~166 ng/shot. The bombardment procedure was performed with a 1000/He Particle Delivery System (Bio‐Rad) using 650 psi rupture disks. After bombardment, embryos rested on osmotic media for 1 hr before being transferred to N6 media. The embryos were analyzed under a Leica M205 FA Stereo Microscope 1–3 days after bombardment.

### 2.6 Fluorescent in situ hybridization (FISH)

T_2_ plants resulting from self‐pollinations were screened via FISH for those in which the transgene was homozygous. These plants were further self‐pollinated to produce lines for perpetuation. FISH was carried out as described for single transgenes (Lamb et al., 2007). The location to chromosome arm in which homozygous transgenes reside is as follows: Cre, 9 long arm (9L); R, 4L; Cre, 9L; phiC31 Integrase, 9L; phiC31 excisionase, 3L.

### 2.7 Materials availability

The five recombinase transformation constructs and the five DsRed test plasmids have been deposited in Addgene (addgene.org). For maize lines with the five transformed recombinases, contact the corresponding author.

## 3 RESULTS

### 3.1 Establishing recombinase expression lines

Each recombinase expression line was established through *Agrobacterium* transformation of Hi‐II immature embryos (Lee & Zhang, 2013) and selected in tissue culture using a bialaphos resistance selectable marker (Figure S1). DNA from leaf tissue of regenerated T_0_ events was analyzed for the presence of the T‐DNA using PCR primer sets that are specific for each recombinase (Table S2). Positive events were crossed to Hi‐II plants to produce heterozygous T_1_ recombinase lines, which were selfed to produce T_2_ homozygous lines. T_2_ plant roots were screened for homozygotes using a single gene detection fluorescent in situ hybridization method (Lamb et al., 2007). The identified lines were selfed to maintain and perpetuate homozygous recombinase stocks. Plants from homozygous recombinase stocks were crossed to Hi‐II tester lines to produce 100% heterozygous immature embryos, which were used in transient reporter assays to determine recombinase functionality in maize.

### 3.2 Experimental design

The strategy to test recombinase functionality in maize cells was adapted from a similar transient assay from Dale & Ow, 1990, in which a construct containing a reporter gene is flanked by oppositely oriented recombinase binding sites and inverted with respect to a constitutive promoter. Here, a *DsRED* gene (*Dicosoma* red fluorescent protein, Bevis & Glick, 2002) is flanked by oppositely oriented sites and inverted with respect to a maize ubiquitin promoter (Ubi) (Figure 2b). Each *DsRED* reporter construct was delivered into a respective recombinase expression line via biolistic bombardment of immature embryos (Figure 2a). If the recombinase is functional, the introduction of a *DsRED* reporter construct will result in an intramolecular inversion reaction between flanking binding sites, which will properly orient the *DsRED* gene with maize Ubi and activate expression (Figure 2c).

### 3.3 Immature embryo bombardment

**Figure 3:**
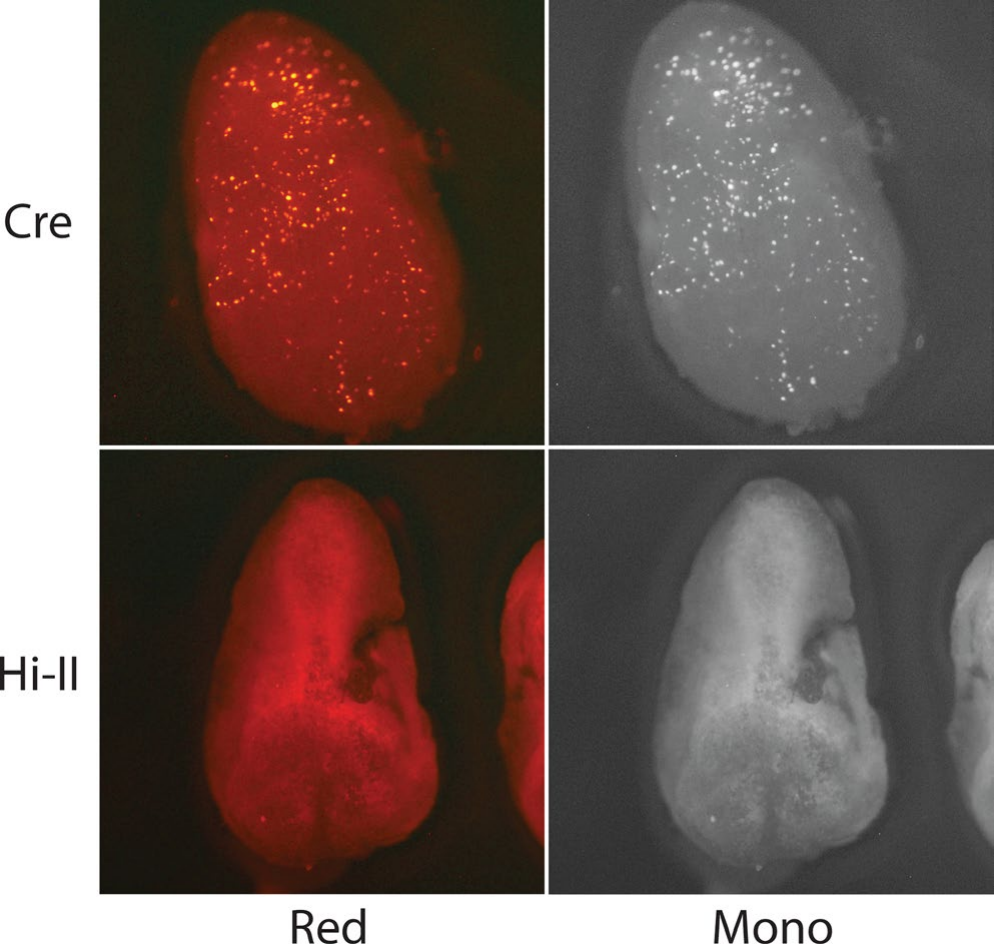
Fluorescent imaging of Cre and Hi‐II embryo bombardment: Top row shows red and monochrome images of Cre‐expressing line immature embryos after bombardment with a *DsRed* reporter construct (Figure 2b). Bottom row shows red and monochrome images of Hi‐II control immature embryos bombarded with Cre *DsRed* reporter construct. Each embryo analyzed was approximately 1.5–2 mm in length

**Figure 4:**
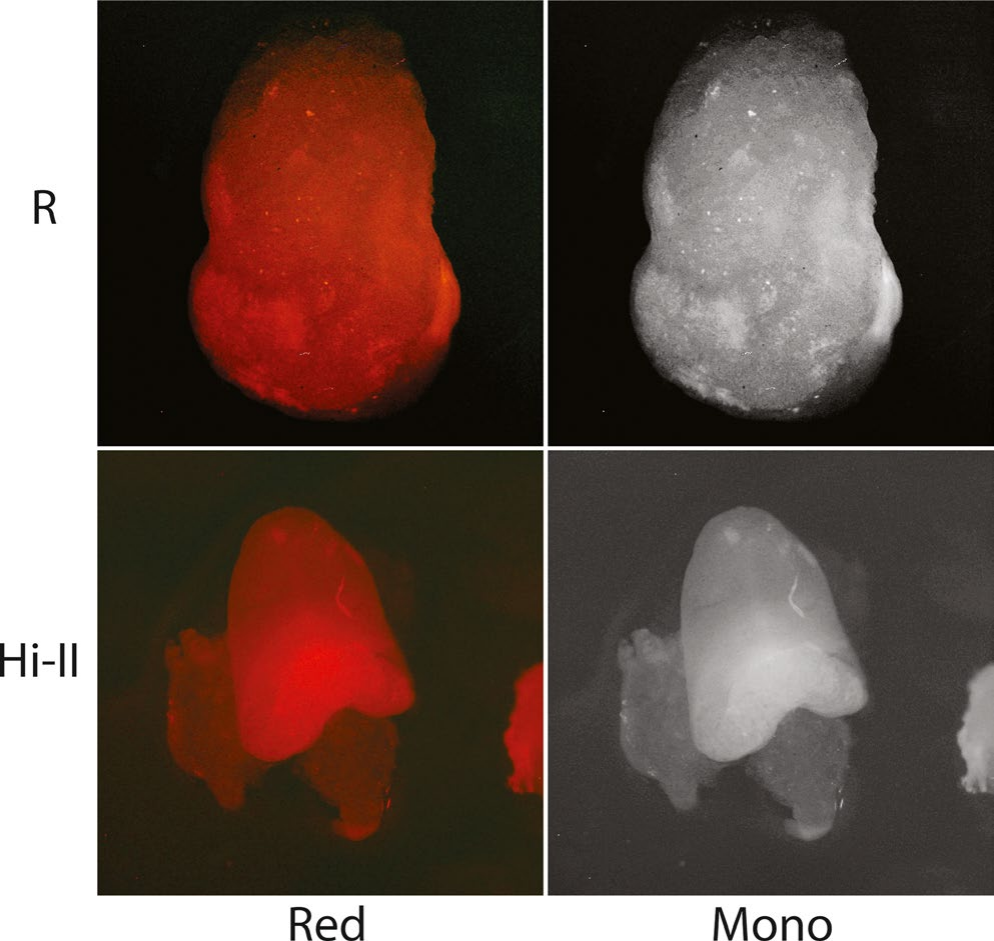
Fluorescent imaging of R and Hi‐II embryo bombardment: Top row shows red and monochrome images of R‐expressing line immature embryos after bombardment with a *DsRed* reporter construct (Figure 2b). Bottom row shows red and monochrome images of Hi‐II control immature embryos bombarded with R *DsRed* reporter construct. Each embryo analyzed was approximately 1.5–2 mm in length

**Figure 5:**
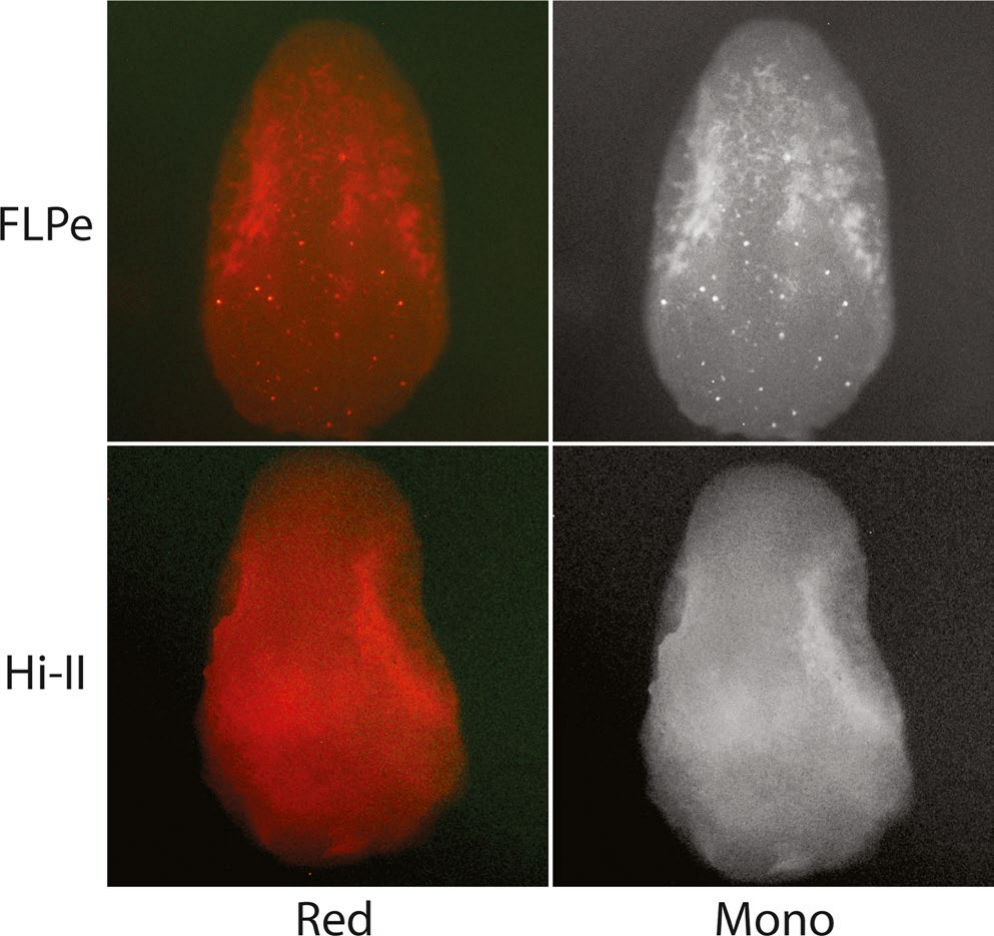
Fluorescent imaging of FLPe and Hi‐II embryo bombardment: Top row shows red and monochrome images of FLPe‐expressing line immature embryos after bombardment with a *DsRed* reporter construct (Figure 2b). Bottom row shows red and monochrome images of Hi‐II control immature embryos bombarded with FLPe *DsRed* reporter construct. Each embryo analyzed was approximately 1.5–2 mm in length

**Figure 6:**
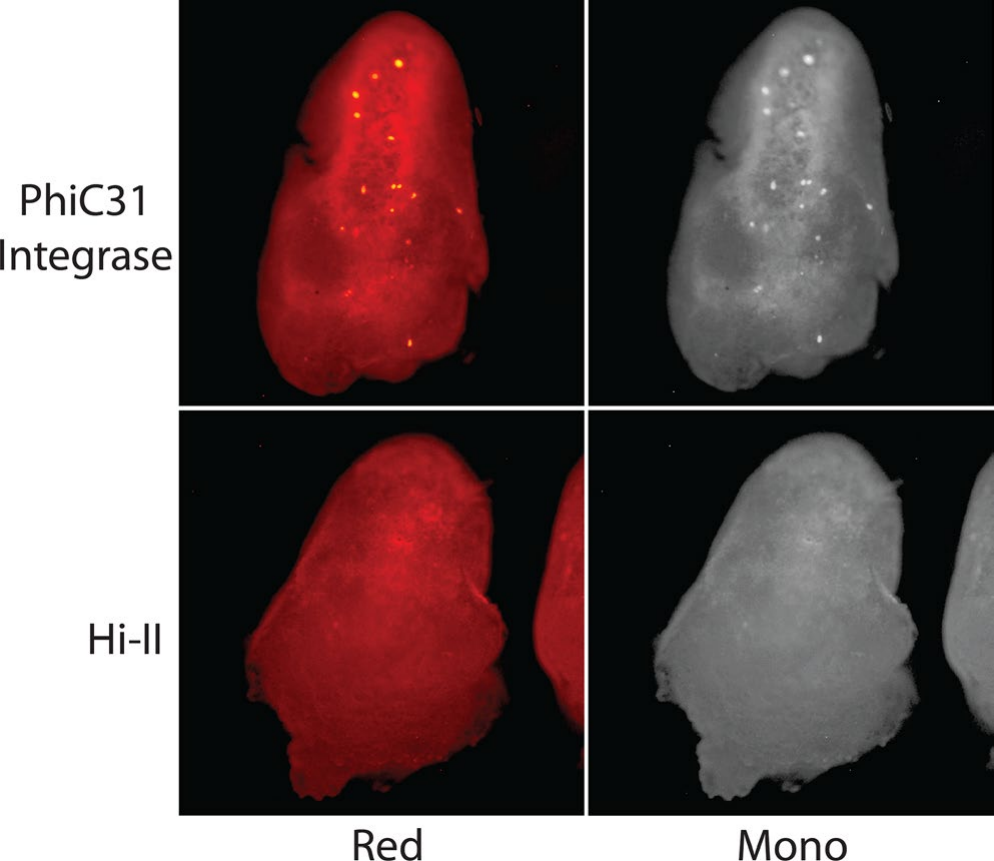
Fluorescent imaging of phiC31 integrase and Hi‐II embryo bombardment: Top row shows red and monochrome images of phiC31 Integrase‐expressing line immature embryos after bombardment with a *DsRed* reporter construct (Figure 2b). Bottom row shows red and monochrome images of Hi‐II control immature embryos bombarded with PhiC31 Integrase *DsRed* reporter construct. Each embryo analyzed was approximately 1.5–2 mm in length

**Figure 7:**
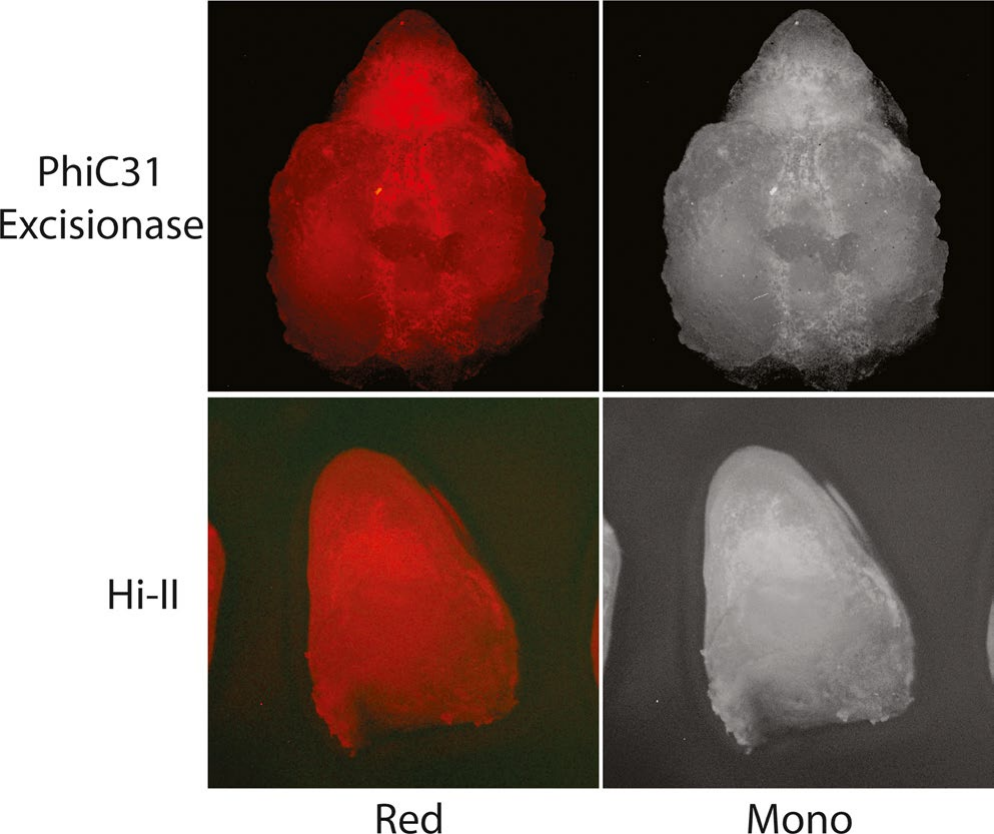
Fluorescent imaging of phiC31 excisionase and Hi‐II embryo bombardment: Top row shows red and monochrome images of phiC31 excisionase‐expressing line immature embryos after bombardment with a *DsRed* reporter construct (Figure 2b). Bottom row shows red and monochrome images of Hi‐II control immature embryos bombarded with PhiC31 excisionase *DsRed* reporter construct. Each embryo analyzed was approximately 1.5–2 mm in length

Recombinase embryos bombarded with *DsRED* reporter constructs were analyzed for signal 24 hr after bombardment under green light excitation (Figures 3, 4, 5, 6, 7). Each stable expression line exhibits red fluorescence “dot” signals on the embryos, a result of the recombinase enzymes binding to respective recombinase binding sites upon delivery of reporter constructs and reorienting *DsRED* to a position that enables expression from the maize Ubi promoter. When comparing these results to Hi‐II control groups bombarded with the same reporter constructs, it is clear that all stable recombinase expression lines are functional in maize.

## 4 DISCUSSION

Site‐specific recombinases are specialized enzymes that naturally function to promote rearrangements of conserved DNA binding sites that are generally under 100 bp in length (Table S1). Due to the size of each sequence, binding sites can easily be incorporated into transformation vectors, enabling precise modifications through the introduction of a respective recombinase system. Strategies for enzyme delivery vary among studies and are dependent on the goals of each research project. While creative methods for expression have previously been demonstrated, such as use of inducible promoters and/or autoexcision methods (Éva et al., 2017; Hoff, Schnorr, & Mundy, 2001; Li et al., 2007; Zhang et al., 2003; Zuo, Niu, Moller, & Chua, 2001), most common approaches are transient or stable expression.

Transient expression relies on the use of *Agrobacterium* or particle bombardment to deliver recombinase coding sequences into host cells containing target sites, leading to a burst of expression that is diminished over time. This enables researchers to make timely modifications of targeted sequences without having to create and maintain stable recombinase expression lines. However, transient expression strategies still utilize plant tissue culture and require a means to select for positive events in the regeneration process. Additionally, it is commonly accepted that recombinase coding sequences are degraded and eventually lost; however, Srivastava and Ow (2001) identified 40% of excision modifications contained a stably integrated Cre recombinase coding sequence after bombardment (Wang et al., 2011). Similarly, Anand et al. (2019) reported the presence of FLP and/or Cre in T_0_ plants when using *Agrobacterium* as the mode of delivery to induce a recombinase‐mediated cassette exchange reaction. Taken together, transient expression of recombinases using *Agrobacterium* or bombardment adds the benefit of timely modifications; however, it requires tissue culture and subsequent screening to obtain a desired plant background.

In the present work, five different recombinase coding sequences were stably integrated into the maize genome through *Agrobacterium*‐mediated transformation. Immature embryos from these lines were subjected to particle bombardment using test constructs containing inverted and inactive *DsRed* sequences (Figure 2b). Upon incorporation into host cells expressing the respective recombinase, *DsRed* coding sequences are reoriented through an inversion reaction and expressed by the maize ubiquitin promoter (Figure 2c). Cells containing both recombinase and target site(s) should fluoresce red under green light excitation, which we observed for each of the five recombinases analyzed (Figures 3, 4, 5, 6, 7). While signal is detectable for each recombinase, it is important to note that the level of fluorescence is different for each enzyme. The easiest explanation for this observation would be enzyme efficiency; however, there are several factors that could account for this difference. These include, but are not limited to, bombardment conditions, binding site copy number, recombinase directionality, and reduced enzyme expression due to insert location in the maize genome. Therefore, the presented lines could be used in a number of genetic engineering applications that involve the need to make precise and predictable site‐specific modifications, including marker gene removal and targeted integrations. The only requirement with these systems is the incorporation of respective recombinase binding sites into target transformation vectors, where the number of different sites and positioning is dependent on the specific application.

Selectable marker excision from stable transgene integrations focuses on the removal of antibiotic or herbicide resistance genes used in the initial establishment of transgenic plants but which are no longer needed. Here, the binding sites are positioned in a “head‐to‐tail” orientation, flanking the marker coding sequence (Figure 1a). In general, recombinase expression lines are created in parallel and used together in a breeding strategy with target lines. Zhang et al. (2003) first demonstrated this method in maize using the Cre/*lox* system and has since then been used with other recombinases, such as wild type or modified versions of FLP from *Saccharomyces cerevisiae* (Kerbach et al., 2005; Li et al., 2009). The reported efficiencies of these FLP variants in monocots is relatively low; however, Akbudak and Srivastava (2011) demonstrated a thermostable version of FLP, enhanced FLP (FLPe), is highly effective in rice. In aspects of ongoing projects, it was demonstrated that the described events for Cre, FLPe, and R can excise sequences from stably integrated transgenes (Cody, 2019).

Transgene targeting to predetermined locations in host genomes has received much attention from the plant research community due to the benefits of increased control and predictability in the transformation process. While most strategies focus on using targeted nucleases to induce double‐stranded breaks (DSB) in a cell with codelivered DNA to foster homologous recombination (HR) or non‐homologous end‐joining (NHEJ), recombinase‐mediated gene targeting utilizes cells previously modified to contain a recombinase coding sequence and a binding site positioned in an optimal location, exhibiting known levels of expression and distinct from endogenous genes. Upon delivery of a transgene containing a respective binding site(s), recombinase enzymes bind to both transgene and target to activate an intermolecular strand‐switching reaction (Figure 1b). Also, in ongoing projects, phiC31 was demonstrated to catalyze integration into a stably transformed target (Swyers, 2019).

The drawback to gene targeting via recombinase enzymes is the need to independently establish recombinase expression and targets lines, which are later crossed together to create cells used in transgene targeting experiments. This is a labor‐intensive and time‐consuming process that requires the use of conventional plant transformation methods and subsequent screening of T_0_ plants. To avoid the additional steps in creating a stable recombinase line, the transient expression may be utilized to carry out desired modifications. However, it is possible that limiting the enzyme expression may decrease the overall targeting efficiency. Indeed, time restrictions may be a reason for increased concentration on methods of HR or NHEJ for gene delivery, since recent advances in targeted nucleases allow quick and precise edits in any known genomic sequence. However, both HR and NHEJ have proven to be inefficient, requiring thousands of embryos for a few events. Additionally, HR seems to restrict the amount of DNA that can be incorporated into target sites. The establishment of stable recombinase expression lines may serve as a solution to researchers who wish to use recombinase enzymes in gene targeting. Here, several different approaches could facilitate efficient integrations into predetermined locations, such as the use of promoter traps in cell cultures or utilization of multiple different recombinases to initiate rearrangements of stable DNA into different locations via breeding. Regardless of the strategy, having a collection of recombinase expression lines will allow the ability to explore this alternative possibility to transgene targeting, which may become powerful tools in the future of genome engineering. While the recombinase transformation cassettes and the test constructs have been shown here to function in maize, the materials should be useful in most plant species.

The future of genetic engineering in plants will most likely use combined engineering strategies allowing researchers to stack a collection of genes efficiently in predetermined genomic locations. With current limitations in publicly available selectable marker genes, this situation at present will require a cyclical strategy of transgene targeting followed by selection removal to prime lines for secondary modifications. This selection recycling strategy was originally presented by Ow (2005) and utilizes the collective activity of many different recombinases and binding sites. Since then, other approaches have been proposed, which also incorporate the use of targeted nucleases to remove previous selection markers (Srivastava, 2019). The recombinase toolkit that we have created may function as foundational material to enable maize researchers to stack a collection of genes in predetermined locations, which will be inherited as a single unit in each successive generation. This toolkit will expand the limits of genome modifications in maize.

## CONFLICT OF INTEREST

JB is a member of the Scientific Advisory Board of Evogene and Ohalo Genetics. NG is currently an employee of Pairwise Plants.

## AUTHOR CONTRIBUTIONS

JC established FLPe and R recombinase expression lines, generated and screened recombinase lines for homozygous backgrounds, created DsRed transient expression test constructs, extracted and bombarded recombinase embryos, analyzed bombarded embryos for DsRed signal on a fluorescent stereo microscope, and wrote the manuscript. NG conceptualized and demonstrated the strategy of using transient GUS expression to show functionality of phiC31 Integrase and phiC31 excisionase in maize, which was later changed to DsRed for increased resolution. CZ provided support and performed *Agrobacterium*‐mediated transformation to establish Cre, phiC31 Integrase, and phiC31 excisionase expression lines. NS provided support and feedback on the manuscript. JB directed the research.

## Supporting information

Supplementary Material

Supplementary Material
