# Supplementary material for: Site‐specific recombinase genome engineering toolkit in maize
Source: Plant Direct. 2020 Mar 9;4(3):e00209. doi: 10.1002/pld3.209 (PMC7061458; doi:10.1002/pld3.209)
Supplement: Supplementary file 1 — Supplementary Material [file PLD3-4-e00209-s001.pdf]

Recombinase - Binding Site	Binding Site Sequence	Directionality
Cre - *lox*	**5′** ATAACTTCGTATAATGTATGCTATACGAAGTTAT **3′**	Bidirectional
R - *RS*	**5′** CGAGATCATATCACTGTGGACGTTGATGAAAGAATACGTTATTCTTTCATCAAATCGT **3′**	Bidirectional
FLPe - *FRT*	**5′** GAAGTTCCTATTCTCTAGAAAGTATAGGAACTTC **3′**	Bidirectional
PhiC31 Integrase	*attB* - **5′** GTGCCAGGGCGTGCCCTTGGGCTCCCCGGGCGCG **3′**<br>*attP* - **5′** CCCCAACTGGGGTAACCTTTGAGTTCTCTCAGTTGGGGG **3′**	Unidirectional
PhiC31 Excisionase	*attL* - **5′** CGGTGCGGGTGCCAGGGCGTGCCCTTGAGTTCTCTCAGTTGGGGGCGTAG **3′**<br>*attR* - **5′** GTAGTGCCCCAACTGGGGTAACCTTTGGGCTCCCCGGGCGCGTACTCCAC **3′**	Unidirectional

Table S1. Recombinase Binding Site Sequence and Directionality: Each recombinase listed is unique in terms of the respective binding sequence, which is between 34 – 58 bp. These sequences can be incorporated into T-DNA molecules to perform site-specific modifications of DNA.

Primer (size bp)	Sequence	T_a
**Cre (447 bp)**	**F - 5′** GAACGTGCAAAACAGGCTCT **3′**<br>**R - 5′** ATCCTTAGCGCCGTAAATCA **3′**	60°C
**R (769 bp)**	**F - 5′** CCAGCGCTCTATTTCCAAGA **3′**<br>**R - 5′** GGCCTCCTTATCCATCTCGT **3′**	60°C
**FLPe (560 bp)**	**F - 5′** GTGAGGGTGAAAGCATCTGG **3′**<br>**R - 5′** AGCACGCTTATCGCTCCAAT **3′**	60°C
**PhiC31 Int (500 bp)**	**F - 5′** CGTGACGATTGTTTCCACTC **3′**<br>**R - 5′** AAGGATTCGCATAACGGTTG **3′**	60°C
**PhiC31 Exc (502 bp)**	**F - 5′** TTACGGCTTCGAGCTTGTTT **3′**<br>**R - 5′** ACGCCTGAAGCTCATACCAC **3′**	60°C

Table S2. Primer Sequences for Screening Recombinase Expression Lines: Each primer set listed is 20 bp in length and designed to anneal to target sequences at 60°C when performing a PCR reaction. The band size in base pairs (bp) of each recombinase is included after the name in parentheses.

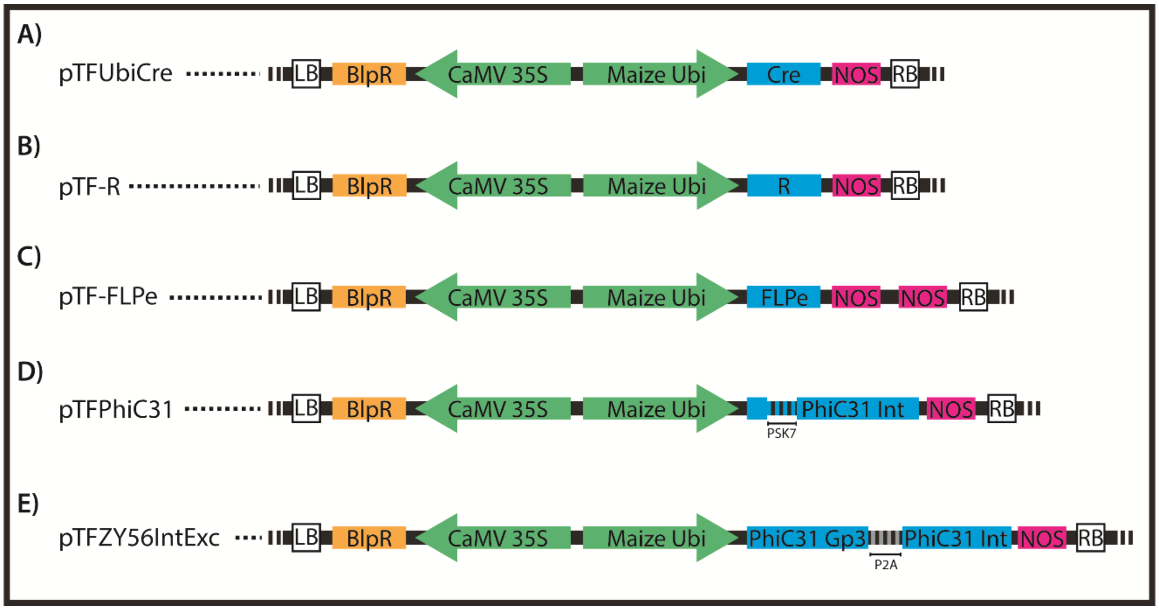

Figure S1. Structure of Recombinase T-DNA Vectors: T-DNA vectors illustrated here are not drawn to scale. All transformation vectors used in this study contain a bialaphos selectable marker (BlpR) under the control of a constitutive cauliflower mosaic virus (CaMV) 35S promoter to select for positive events in plant tissue culture conditions. **(A)** Cre expression is driven by a maize ubiquitin 1 (Ubi1) promoter and terminated by a nopaline synthase termination coding sequence (Nost). **(B)** R expression is controlled by maize Ubi1 and terminated by Nost. **(C)** FLPe is expressed by a maize Ubi1 promoter and terminated by two successive Nost coding sequences. **(D)** pTFPhiC31 expression is controlled by maize Ubi1 and terminated by Nost. PhiC31 Integrase coding sequence contains an intron derived from the *Petunia hybridia Psk7* gene, which helps increase recombinase expression. **(E)** pTFZY56IntExC contains the coding sequences for the excisionase modifying protein (Gp3) and integrase, which are fused by a P2A cleaving peptide. P2A enables the coexpression of Gp3 and integrase using a single maize Ubi1 promoter.
