# Supplementary material for: Site‐specific recombinase genome engineering toolkit in maize
Source: Plant Direct. 2020 Mar 9;4(3):e00209. doi: 10.1002/pld3.209 (PMC7061458; doi:10.1002/pld3.209)
Supplement: Supplementary file 2 — Supplementary Material [file PLD3-4-e00209-s002.pdf]

Response to the Reviews:

We thank the reviewers for their helpful comments.

Reviewer #1:

The authors established lines expressing several different Recombinases and bombarded immature embryos from these lines with the inverted DsRed constructs that are flanked by the appropriate recombinase binding site. If Recombinases are active, it will flip the DsRed to the correct orientation that will allow fluorescence. Their control was bombarding DsRed into immature embryos w/o recombinases to establish very low level (or not detectable) fluorescence. The data indicates that this approach does work to varying degrees. Performing expression analysis of the recombinases in immature embryos would have greatly improved this paper (especially for the R-expressing line). To the authors credit, this and other deficiencies are mentioned in the discussion, thus they didn't read too much into their data and made appropriate conclusions.

Other notes: I found the paper to be well written. I did find the misspelling of restriction on page 6, second paragraph. Also, check citations as I could not find Lamb et al (on page 9) in the citations listed at the end of the paper.

Response: The recombinases Cre, FLPe, R and Int have been shown to work in other circumstances in ongoing work, which was cited via Ph. D. theses. It would be complicated to make extensive explanations of these experiments, which are not yet at a conclusion. We have corrected the spelling and citation issues raised.

Reviewer #2:

In this manuscript Cody and colleagues describe a collection of maize lines with stably integrated transgenes expressing 5 different recombinases. Genetic engineering in plants, and particularly crops, is a critical tool for exploring basic biological functions and for executing biotechnological breeding strategies. The availability of maize lines expressing these popular recombinases will certainly be useful to the community, so the manuscript is both novel and significant.

Major Comments

1. The authors establish a set of stably integrated transgenic maize lines that express either Cre, R, FLPe, phiC31 Integrase, or phC31 Excisionase. These are envisioned as tools for future application by the maize community. Among their possible uses is in crosses with lines that have transgenes with recombinase recognition sites. In these kinds of experiments it would be helpful to know the genomic location of the recombinase transgene. This could be accomplished by PCR-based sequencing of the DNA flanking the transgene insertion sites.

2. In the "Establishing Recombinase Expression Lines" section it would be very helpful to provide a description (perhaps a table) of how many stable lines were recovered for each of the recombinase constructs. Following on from comment #1, it would be helpful for

future users to be able select different lines with different insertion sites.
3. Representative images of transient transformation of the DsRed reporter construct into each of the stale lines demonstrates that the recombinases are functional. However, as noted in the discussion, the lines show significant differences in apparent efficiency (i.e. it appears Cre > PhiC31 Integrase > FLPe > R > PhiC31 Excisionase). The authors argue that this could be due to actual differences in the biological activities of the recombinases, or to secondary issues like position effect. It would be helpful for future users to try to differentiate between those possibilities. If more than one line for each recombinase was recovered statistical analysis of the apparent efficiency (as measured by DsRed foci numbers) could be compared.
4. Since this is being pitched as a toolkit that will be useful to the broader maize community, it would be nice to explicitly state in the manuscript how interested users can access the lines (will they be deposited in a stock center?), similarly it would be really helpful if the sequence of the recombinase constructs are deposited in a database and the constructs themselves made available through a repository like Addgene (this would help folks make similar resources in other genetic backgrounds).

Minor Comments

1. There are a number of spelling and English usage issues (for example, tumefaciens is misspelled in the introductory paragraph, but there are also many other mistakes). The manuscript should be very carefully proofread before resubmission.
2. The color/shape scheme used in Figure 1 should be better described in the figure legend.

Summary

The manuscript is a succinct description of a useful set of tools for maize molecular geneticists. There are no technical flaws, but there are some relatively easy things (described above) that the authors could do to make the resource more useful to future users.

Response: The chromosome arm on which each recombinase resides was determined by FISH and is now noted in the Materials and Methods section of the revision. We have only a single transformation event of each of the recombinases. We developed these lines for other purposes and so we sought a line with functionality and moved on. While that is the case, we see the utility of these materials for others in the maize community and indeed for any other plant species because now that the collection of constructs is assembled and available, it can be transformed in other model species as well. We have deposited the constructs for all five recombinases and their test constructs in Addgene, which is now noted in the manuscript in Materials and Methods. We contacted Marty Sachs, the director of the Maize Stock Center, about the possibility of accepting these materials. While the answer was not a categorical "No", the stock center will not propagate them and we would have to supply a quantity that we do not currently have. Moreover, the Coop will not complete APHIS permits for interstate transfer of transgenic materials (an issue that can be overcome by providing them with the information for the recipient to apply) nor handle any materials for which the recipient wants to complete an Material Transfer Agreement (MTA). In the end, we tabled the discussion and have placed in the manuscript that the maize lines are available upon request from the corresponding author. We have no IP on these materials. In our experience some institutions still want an MTA on materials even

though we have no IP on them. If that is the case, we can have our Technology Transfer prepare one. With the increasing ease with which transformation of different maize inbreds can be achieved, the availability of the transformation constructs at Addgene is the more important aspect of material availability. We have corrected the misspellings in the manuscript and have expanded the legend to Figure 1 as suggested.

--- Editor comments:
Both reviewers were very favorable and see the importance and utility of these lines for the maize community. Beyond typographical errors, there are a few small changes needed:

1. As suggested by reviewer 2, could any information about the location of the insertions be included in the manuscript? It is not necessary to identify the insertion site precisely, but if you have any information regarding rough map position, please include this in the manuscript.

2. Similarly, if there are multiple stable lines available, please provide this information.

3. It is important that the manuscript describe how the community can access the transgenic lines and recombinant constructs. Reviewer 2's suggestion to deposit the plasmids at Addgene is a good option, although not necessary.

Response: We agree that these are valuable points and have supplied this information in the revision in the Materials and Methods section. As noted above, all of the constructs have been sent to Addgene and the maize lines can be obtained from the corresponding author. Both points have been added to the manuscript.
